# No association between intravenous fluid volume and endothelial glycocalyx shedding in patients undergoing resuscitation for sepsis in the emergency department

**DOI:** 10.1038/s41598-022-12752-x

**Published:** 2022-05-24

**Authors:** Stephen Macdonald, Erika Bosio, Nathan I. Shapiro, Lois Balmer, Sally Burrows, Moira Hibbs, Thomas Jowitt, Lisa Smart, Glenn Arendts, Daniel Fatovich

**Affiliations:** 1 Centre for Clinical Research in Emergency Medicine, Harry Perkins Institute of Medical Research, Royal Perth Hospital, Level 6, Q Block, PO Box 2213, Perth, WA6000 Australia; 2 Medical School, University of Western Australia, Perth, Australia; 3 Emergency Department, Royal Perth Hospital, Perth, Australia; 4 Beth Israel Deaconess Medical Centre, Harvard University, Boston, USA; 5 Centre for Precision Health, School of Medical and Health Sciences, Edith Cowan University, Joondalup, Perth, WA Australia; 6 Royal Perth Hospital Research Foundation, Perth, Australia; 7 Research Centre, Royal Perth Hospital, Perth, Australia; 8 School of Science, Health, Engineering and Education, Murdoch University, Perth, Australia; 9 Emergency Department, Fiona Stanley Hospital, Perth, Australia

**Keywords:** Infectious diseases, Immunopathogenesis, Biomarkers

## Abstract

Endothelial glycocalyx (EG) shedding is associated with septic shock and described following intravenous (IV) fluid administration. To investigate the possible impact of IV fluids on the pathobiology of septic shock we investigated associations between biomarkers of EG shedding and endothelial cell activation, and relationships with IV fluid volume. Serum samples were obtained on admission (T0) and at 24 h (T24) in patients undergoing haemodynamic resuscitation for suspected septic shock in the emergency department. Biomarkers of EG shedding—Syndecan-1 (Syn-1), Syndecan-4 (Syn-4), Hyaluronan, endothelial activation—Endothelin-1 (ET-1), Angiopoeitin-2 (Ang-2), Vascular Endothelial Growth Factor Receptor-1(VEGF-1) and leucocyte activation/inflammation—Resistin, Neutrophil Gelatinase Associated Lipocalin (NGAL) and a marker of cardiac stretch—Pro-Atrial Natriuretic Peptide (Pro-ANP) were compared to the total IV fluid volume administered using Tobit regression. Data on 86 patients (52 male) with a mean age of 60 (SD 18) years were included. The mean fluid volume administered to T24 was 4038 ml (SD 2507 ml). No significant association between fluid volume and Pro-ANP or any of the biomarkers were observed. Syn-1 and Syn-4 were significantly correlated with each other (Spearman Rho 0.43, *p* < 0.001) but not with Hyaluronan. Syn-1 and Syn-4 both correlated with VEGFR-1 (Rho 0.56 and 0.57 respectively, *p* < 0.001) whereas Hyaluronan correlated with ET-1 (Rho 0.43, *p* < 0.001) and Ang-2 (Rho 0.43, *p* < 0.001). There was no correlation between Pro-ANP and any of the EG biomarkers. Distinct patterns of association between biomarkers of EG shedding and endothelial cell activation were observed among patients undergoing resuscitation for sepsis. No relationship between IV fluid volume and Pro-ANP or any of the other biomarkers was observed.

## Introduction

Intravenous (IV) fluid resuscitation is the first line treatment for the haemodynamic resuscitation of patients with septic shock, with international consensus guidelines recommending initial resuscitation with 30 ml/kg of isotonic crystalloid over the first three hours^1^. There is emerging evidence of harm associated with excess fluid balance in sepsis patients^2–4^ and clinical trials in some populations have demonstrated better patient outcomes with a fluid-restricted approach^5,6^. Consequently, there is clinical uncertainty about the optimal dosing of IV fluid and the associated timing of vasopressor initiation^7^, and there is substantial variation in practice^8^.

One suggested mechanism for harm with IV fluids is shedding of the endothelial glycocalyx (EG) layer due to hypervolaemia and/or haemodilution^9,10^. The EG is a 0.5 μm thick gel-like layer of proteoglycans anchored to the luminal endothelial surface, with glycosaminoglycan (GAG) molecules incorporated throughout the structure. Syndecans are membrane-bound proteoglycans on the apical surface of endothelial cells to which several different GAGs such as Heparan Sulfate and Chondroitin Sulfate attach covalently to form the EG layer. In contrast, the GAG Hyaluronan is not attached to Syndecan but to cell surface CD44 or other GAG molecules^11–13^. In settings such as inflammation, cleavage of the Syndecan ectodomain by sheddases such as matrix metalloproteinases (MMPs) delivers the soluble component to the circulation^14^.

The EG is a key determinant of fluid homeostasis as well as maintaining inflammatory and haemostatic quiescence in health^15^. EG shedding occurs in response to both acute and chronic inflammation and elevation of blood Syndecan-1 (Syn-1) and Hyaluronan concentration has been described in patients with sepsis^16,17^. It has been suggested that EG shedding leads to fluid extravasation and propagation of the systemic inflammatory response in sepsis with resultant worsening organ dysfunction and shock^9,18–20^. However, recent preclinical studies have challenged whether EG shedding results in increased vascular permeability^21,22^. Additionally, it is unclear whether exogenous fluids have a direct effect on the EG, or whether this is mediated via MMPs activated by natriuretic peptides released in response to cardiac chamber dilatation^9,23,24^.

While it is well established that increased biomarkers of EG degradation are associated with illness severity and mortality in sepsis, data from patients undergoing fluid resuscitation have shown variable associations between IV fluid volume and concentrations of individual EG biomarkers^17,25,26^. Further, studies in healthy subjects and perioperative settings may not be relevant in settings such as sepsis where EG shedding is already established^27^. The question of the incremental effect of exogenously administered fluids on an already injured EG is unresolved.

While clinical uncertainty remains, it is important to test the hypothesis that IV fluid volume is detrimental in sepsis via the effect of EG shedding. The objective of this study was to examine the relationship between the volume of IV fluid administered during the first 24 h from commencement of treatment in the emergency department (ED) and soluble biomarkers of EG shedding, endothelial activation and inflammation in adult patients with suspected septic shock. The secondary objective was to explore associations between EG biomarkers and biomarkers of endothelial cell activation and inflammation after fluid resuscitation.

## Methods

### Participants and setting

We undertook secondary analysis of a convenience sample of adult (age 18 years or older) ED patients undergoing haemodynamic resuscitation for suspected septic shock recruited into two observational studies one in Perth, Australia between 2010 and 2015, the other in Boston, USA between 2012 and 2018. The methodologies of each study have previously been described^28,29^. The inclusion criteria were (1) the presence two or more systemic inflammatory response (SIRS) criteria and (2) a clinical decision to administer IV antibiotics for suspected infection and (3) a systolic blood pressure of < 90 mmHg despite at least 1 L of IV fluid along with either (a) blood lactate > 2 mmol/L or (b) a requirement for vasopressors to maintain perfusion targets. These criteria reflect an operational definition for septic shock. Recruitment took place according to the Sepsis 2 criteria which were based upon SIRS. The Sepsis 3 criteria introduced in 2016 placed greater importance on lactate and vasopressor requirement to define septic shock. All of these patients were undergoing resuscitation for hypotension/hypoperfusion related to infection in the ED and thus represent a clinically relevant population in terms of the research question.

### Data collection

Blood samples were collected on recruitment in the ED (T0), and subsequently 24 h later (T24). Samples were processed within 2 h of collection, with serum collected by centrifugation at 3000 rpm for 10 min, followed by storage of 0.5 ml aliquots at − 80 °C. Clinical data included baseline demographics, vital signs, laboratory results, suspected source of infection, Sequential Organ Failure Assessment (SOFA) scores, Charlson Comorbidity Score (CCS), admission to the intensive care unit (ICU), length of hospital stay and in-hospital mortality. Fluid volumes were calculated as the total administered IV, prior to each time point (crystalloid, colloid, blood products), including prehospital fluids, but excluding drug infusions or flushes.

### Biomarker analyses

Biomarker analyses were determined by enzyme-linked immunosorbent assay (ELISA). Selected EG biomarkers were Syndecan-1 (Syn-1), Syndecan-4 (Syn-4) and Hyaluronan. Selected endothelial cell activation biomarkers measured were Endothelin-1 (ET-1), Angiopoietin-2 (Ang-2) and Vascular Endothelial Growth Factor Receptor-1 (VEGFR-1). We also measured a biomarker of cardiac chamber stretch, Pro-Atrial Natriuretic Peptide (Pro-ANP), and the leucocyte activation biomarkers Resistin and Neutrophil Gelatinase-Associated Lipocalin (NGAL). These biomarkers were selected based upon previous work by our groups^30–32^. All analytes except ET-1 were assayed using R&D ‘DuoSet’ Enzyme Linked Immunosorbent Assays (ELISA), (R&D Systems Inc., MN, USA). Each kit was individually optimised prior to use, to achieve average intraplate coefficient of variations (CVs) as follows: Syn-1 5.14%, Syn-4 4.39%, Hyaluronan 8.59%, Ang-2 5.40%, VEGFR-1 8.31%, Pro-ANP 5.68%, Resistin 5.09% and NGAL 4.58%. ET-1 was assayed using a fully validated Quantikine ELISA kit (R&D Systems, MN, USA) in accordance with manufacturer’s instructions. All biomarker analyses were undertaken in the laboratory of the Centre for Clinical Research in Emergency Medicine in Perth, Australia.

### Statistical analysis

We measured the relationship between total cumulative fluid volume and biomarkers at T0 and T24 and the change in value of each biomarker between T0 and T24, and with the cumulative total volume at T24. The relationship between pairs of biomarker concentrations at T24 was analysed by Spearman correlation. Because of censoring of some biomarker results both below and above the limit of assay detection, repeated measures Tobit regression was employed with an interaction of time and fluids to test for an association between cumulative fluid volume at T24 and the change over time in the biomarker. For biomarker variables with no censored values, random effects linear mixed models were used. Regression analyses are dependent on assumptions, but these are specific to each regression not to the data set as a whole. All biomarker concentrations were log transformed for multiple reasons. The first is that none of the biomarkers were normally distributed (for censored distributions this relates to the values between the limits) and while this is not an assumption of regression, but rather normality of the residuals, the latter generally occurs if the former is true. Secondly the log transformation can often solve the problem of heteroscedasticity if present. When the transformation did not successfully address these issues, bootstrapping was employed. This resampling technique produces standard errors and p values that are robust to the violation of assumptions.

Associations between the cumulative fluid volume and biomarker concentrations were examined for possible non-linearity using scatterplots with a Lowess fit as well as multivariable regression spline models. Where non-linearity was detected, piecewise Tobit or linear regressions were used to estimate multiple slopes in the one model. All regression models were adjusted for age, sex, baseline mean arterial pressure, baseline lactate, SOFA score, CCS, suspected source of infection (respiratory, urinary, other) and recruitment site. Where violations of assumptions were detected, regression analyses were boot-strapped to produce robust standard errors and p values. The analyses were performed using Stata v15 (College Station, TX, USA). Given the exploratory nature of the study p values were not adjust for multiple testing.

### Ethics approval and consent to participate

The study was approved by the Human Research Ethics Committee of Royal Perth Hospital and the Institutional Review Board of Beth Israel Deaconess Medical Center. Informed consent was obtained from the participant or their next of kin, or under a waiver of consent for low-risk observational research in accordance with local laws.

## Results

Of 86 adult participants recruited, 11 (13%) died within 30 days. The characteristics of the participants are described in Table 1.

**Table 1:** Participant characteristics.

N	86
Age (years)	64 (18)
Males *N* (%)	52 (60%)
Temperature ^O^C	37.4 (1.6)
Heart rate (bpm)	101 (25)
Respiratory rate (bpm)	24 [20, 28]
Mean arterial blood pressure (mmHg)	65 [58, 79]
Lactate (mmol/L)	2.7 [1.7, 3.6]
Charlson comorbidity score (*N* = 74)	3 [1, 5]
SOFA Score	8 (4)
**Source of infection *N* (%)**	
Respiratory	35 (41%)
Urinary	17 (20%)
Other	34 (39%)
Admitted to ICU	65 (76%)
Invasive ventilation	26 (43%)
Died in hospital	11 (13%)

Values are *n* (%), mean (sd) or median [first to third quartile: Q1, Q3]. *SOFA* Sequential Organ Failure Assessment.

The mean cumulative total fluid volume administered prior to T0 was 2330 ml (SD 1698 ml) and to T24 was 4073 ml (SD 2507 ml). A vasopressor infusion was commenced in 55 (64%) participants within 24 h. The predominant fluid administered was isotonic crystalloid. Additionally, fourteen patients received colloid (12 synthetic gelatin solution, 2 albumin) and one received blood. Two participants received more than 10L of IV fluid (12L and 14L respectively) within the first 24 h.

The median concentration of each biomarker at the two sampling time points are shown in Table 2. Syn-1 and Ang-2 significantly increased over time, whereas Syn-4, ET-1 and NGAL significantly decreased over time. There were no significant differences detected between time points for the remaining biomarkers. No significant difference in the EG shedding biomarkers (Syn-1, Syn-4 and Hyaluronan) was found at either time point between those with SOFA Renal < 3 vs >  = 3, or across source of infection groups (respiratory, urinary, other). Both Syn-1 and Hyaluronan were significantly higher among those admitted to ICU at both time points, compared to those not admitted to ICU, but not between those who were ventilated and those who were not (Supplementary data file).

**Table 2:** Biomarker results.

	T0	T24	*P* value
Syndecan-1 (pg/ml)	8684 [4412, 20883]	10,563 [5600, 23460]	0.001
Syndecan-4 (pg/ml)	794 [370, 1942]	629 [193, 1521]	0.002
Hyaluronan (ng/ml)	168 [64, 474]	223 [48, 527]	0.64
VEGFR-1 (pg/ml)	479 [125, 2594]	732 [125, 24561]	0.6
ET-1 (pg/ml)	3.6 [2.4, 5.6]	2.6 [1.9, 3.6]	< 0.001
Ang-2 (pg/ml)	3752 [2610, 8939]	5409 [2610, 10256]	0.03
Pro-ANP (pg/ml)	4162 [2162, 13122]	4496 [2218, 8152]	0.57
Resistin (ng/ml)	105 [54, 227]	119 [46, 232.4]	0.21
NGAL (ng/ml)	1230 [690, 2114]	1094 [492, 1979]	0.008

Values are medians [first to third quartile: Q1, Q3]. *VEGFR* Vascular Endothelial Growth Factor Receptor, *ANP* Atrial Natriuretic Peptide, *NGAL* Neutrophil Gelatinase-Associated Lipocalin. *P* values were obtained from unadjusted random effects Tobit or linear mixed models.

**Figure 1:**
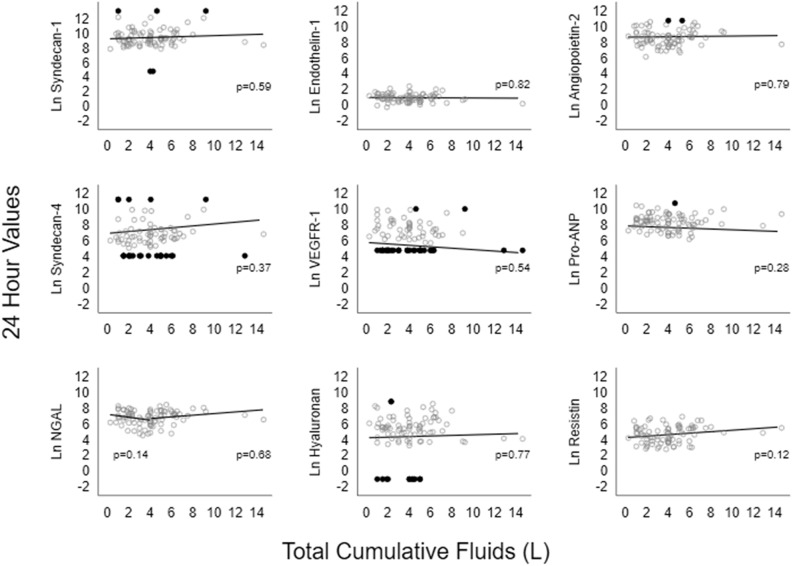
Relationship between volume of intravenous fluid and each biomarker at T24 adjusted for age, sex, mean arterial blood pressure, lactate, Charlson score, SOFA score, source of infection and recruitment site. Circles – data points, Dots – censored values.

**Figure 2:**
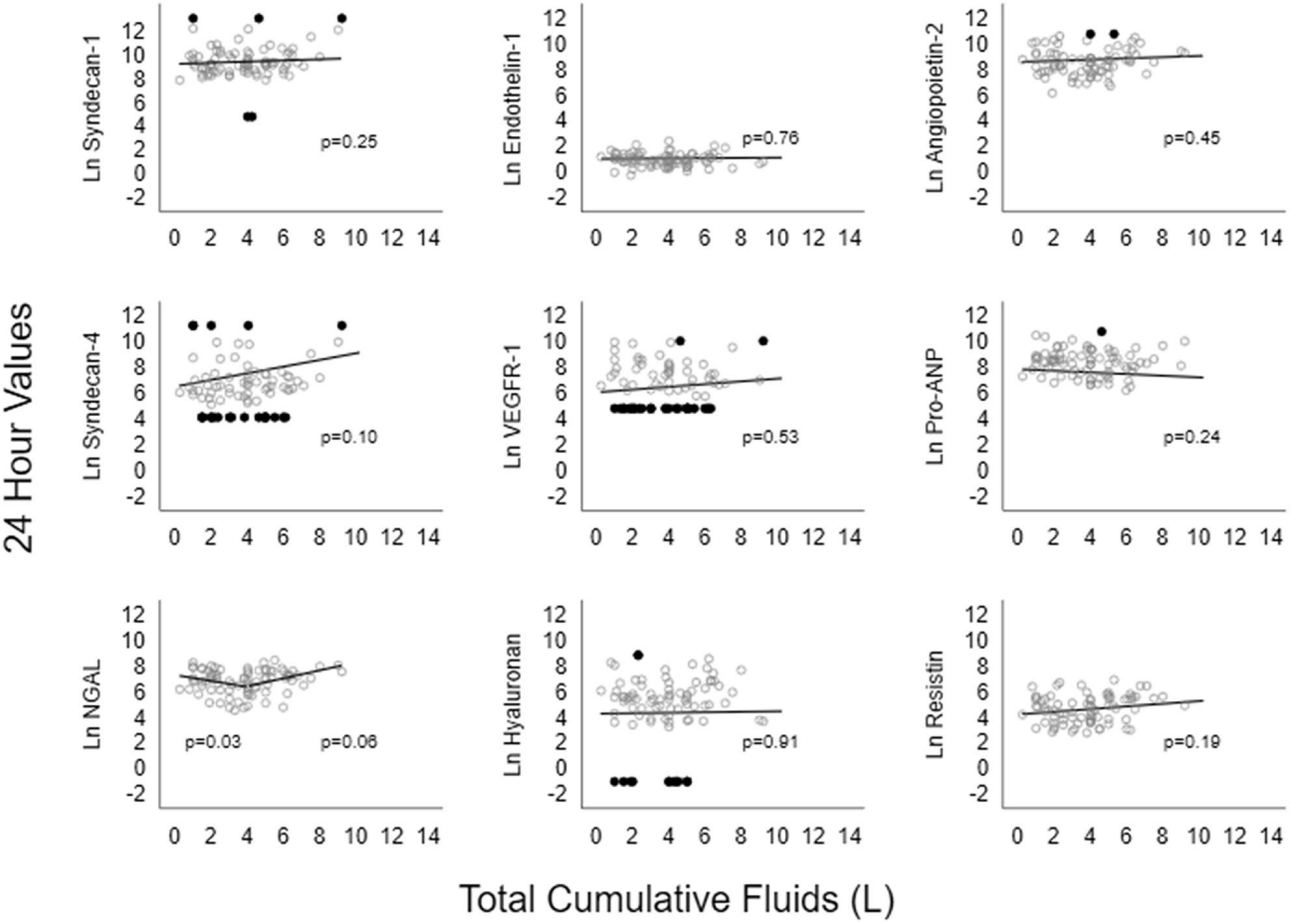
Sensitivity analysis (excluding two outlier cases) of relationship between volume of intravenous fluid and each biomarker at T24 adjusted for age, sex, mean arterial blood pressure, lactate, Charlson score, SOFA score, source of infection and recruitment site. Circles – data points, Dots – censored values.

No significant relationship was observed between the biomarkers and the cumulative fluid volumes at T0 or at T24. No significant interaction between fluid volume at T24 and the change over time was found for any of the biomarkers. Figure 1 demonstrates the relationship between the value of each biomarker at T24 and the cumulative total fluid volume, adjusted for baseline confounding variables. Following the removal of two outlier values of fluid volume, a sensitivity analysis was conducted to determine whether these were masking a potential association between fluid volume and the measured biomarkers (Fig. 2). Again, there was no significant association found between any of the biomarkers and the fluid volume administered.

Table 3 shows the results of Spearman correlations between each of the biomarkers at T24. Syn-1 and Syn-4 were significantly correlated with each other but not with Hyaluronan. Syn-1 and Syn-4 both correlated with VEGRF-1 whereas Hyaluronan correlated with ET-1 and Ang-2 Hyaluronan was also weakly correlated with the inflammatory biomarkers, Resistin and NGAL. Similar patterns were observed for biomarkers measured at T0 (data not shown). There was no correlation between Pro-ANP and any of the EG biomarkers.

**Table 3:** Correlations between biomarkers at T24.

	Syn-1	Syn-4	Hyaluronan	VEGFR-1	Endothelin-1	Ang-2	Pro-ANP	Resistin	NGAL
Syn-1	1								
Syn-4	0.43 (< 0.001)	1							
Hyaluronan	0.15 (0.21)	− 0.05 (0.7)	1						
VEGFR-1	0.56 (< 0.001)	0.57 (< 0.001)	0.11 (0.38)	1					
Endothelin-1	0.19 (0.11)	0.13 (0.29)	0.43 (< 0.001)	0.32 (0.006)	1				
Ang-2	0.16 (0.18)	0.08 (0.51)	0.43 (< 0.001)	0.23 (0.05)	0.47 (< 0.001)	1			
Pro-ANP	0.18 (0.13)	0.12 (0.29)	0.20 (0.10)	0.21 (0.08)	0.09 (0.44)	0.11 (0.33)	1		
Resistin	0.21 (0.07)	0.07 (0.53)	0.37 (0.001)	0.07 (0.57)	0.25 (0.04)	0.47 (< 0.001)	0.21 (0.08)	1	
NGAL	0.2 (0.1)	− 0.05 (0.69)	0.32 (0.006)	0.20 (0.09)	0.27 (0.02)	0.50 (< 0.001)	0.35 (0.002)	0.42 (< 0.001)	1

Spearman’s Rho (p).

*Syn* Syndecan, *VEGFR* Vascular Endothelial Growth Factor, Ang, *ANP* Atrial Natriuretic Peptide, *NGAL* Neutrophil Gelatinase Associated Lipocalin.

## Discussion

### Statement of principal findings

In this observational study of adult patients with clinically suspected septic shock undergoing fluid resuscitation in two emergency departments, no relationship between volume of intravenous fluid and concentrations of biomarkers of EG shedding or endothelial activation was found. We did observe two distinct patterns of potential EG shedding; a significant association between Syn-1/Syn-4 and VEGFR-1; and between Hyaluronan and Ang-2, ET-1, Resistin and NGAL. There was no observed correlation between Syn1/Syn4 and Hyaluronan, nor between Pro-ANP and any of other EG biomarkers.

### Strengths and weaknesses of the study

Strengths of the study include recruitment during the initial resuscitation phase of suspected septic shock. Limitations include its observational design which, despite controlling for variables that would be expected to influence the volume of fluid administered, residual confounding cannot be discounted. The large number of censored values for Hyaluronan (22/172 censored observations at T24) and VEGFR-1 (71/166 censored observations at T24) reduced the power to detect significant differences for these biomarkers. Measurements were limited to two time points at T0 and T24 and therefore would not capture the biomarker profiles during the first 6 h of resuscitation when the largest volumes of fluid are typically administered. The nature of real-world clinical research among acutely sick patients in the emergency setting is that many patients had treatment initiated prior to enrolment, even pre-hospital. The analyses chosen take account of this by describing the fluid administered prior to T0. The biomarkers selected were not a comprehensive suite of all molecules released by EG shedding and endothelial cell activity^27^. Moreover, soluble biomarkers are a surrogate measure of EG shedding and their utility as an accurate quantitative measure of this process has been questioned in a recent literature review^27^. The impact upon the expression of EG from vasopressors is unclear. In mitigation of this, the SOFA score does capture the use of vasopressors, and we adjusted for SOFA in the regression analyses. Due to the retrospective nature of this analysis, we were unable to perform direct estimation of EG thickness (for example by intravital sublingual microscopy) which would have provided a more comprehensive assessment^33^. Finally, the small number of participants precluded any exploration of potentially important differences in the effect of fluid types on the EG (*e.g.,* balanced vs unbalanced crystalloids, synthetic colloids, or albumin)^15^ and patient subgroups. Given these factors, the lack of any demonstrated positive associations between fluid volume and EG shedding does not necessarily imply that such associations do not exist.

### Strengths and weaknesses in relation to other studies

We did not observe any association between IV fluid volume, Pro-ANP or any biomarker of EG shedding, endothelial activation of inflammation. These clinical observations are in contrast to experimental and preclinical studies which have found evidence of EG shedding associated with administration of exogenous fluids, with some studies also showing a concurrent rise in Pro-ANP^10,18,34^. In patients with sepsis in the ED, Smart et al.described a positive association between fluid volume and hyaluronan during the first 3 h of resuscitation however no such relationship was seen for Syn-1 or Syn-4^17^. Hippensteel et al.reported an association between the volume of IV fluid and the expression of Heparan Sulfate (measured by mass spectroscopy) after 6 h of resuscitation in patients with sepsis^25^. The timing of sampling in our study prevents direct comparison with these studies.

In a randomised trial comparing a fluid restricted resuscitation strategy versus a liberal fluid regimen for resuscitation among patients with septic shock, Soaraya et al.found Syn-1 levels at 6 h to be lower with the limited fluid regimen (geometric mean ratio of 0.82 (95% CI 0.66–1.02), p = 0.07)^26^. In a multicentre ICU study of 619 patients there was no relationship between fluid volume in the first day of admission and Syn-1 among patients with sepsis, although Syn-1 was associated with illness severity and mortality^35^. Similarly, in a prospective study of 175 patients with sepsis, Puskarich et al.found that high Syn-1 levels were associated with increased renal injury and mortality but there was no significant difference in the volume of IV fluids administered between two groups categorised according to the level of Syn-1^36^. In contrast, Soraya et al. found that Syn-1 measured at the time of admission to the ICU was associated with subsequent fluid requirements, as well as with organ failure and mortality^37^. This raises the question of whether an association between elevated EG biomarkers and fluid volume simply represents larger fluid resuscitation requirements in patients with a leaky vasculature rather than fluid induced EG shedding. Further complicating the issue are recent preclinical experimental studies which have challenged whether there is any relationship between EG shedding and increased vascular permeability^21,22,38^.

The hypothesis of a relationship between fluid volume and EG degradation is supported by a study by Pouska et al. (*n* = 66) which demonstrated lasting changes in the perfusion boundary region (a surrogate measure of EG thickness assessed by intravital sublingual microscopy) following IV volume loading in sepsis patients^39^. There was a transient increased in PBR in elective surgical patients which resolved. A similar effect was seen in septic shock patients, however this persisted at least 120 min after the infusion. Whether this is a direct effect or mediated by the effect of ANP on MMPs is not clear^9^. Our results are consistent with those of Hippensteel et al.who found no significant relationship between ANP and Heparan sulfate, interleukin-6 levels (a marker of acute inflammation) or mortality^25^. This, however, may be explained by timing of sampling in different studies. Belavic et al. demonstrated an early rise in ANP along with Syn-1 after administration of IV fluids in surgical patients^40^. Possible explanations for these discordant results could be due to insufficient volume administered to generate cardiac stretch to increase expression. Alternatively, unlike euvolemic perioperative patients, sepsis patients may have absolute or relative hypovolaemia at baseline.

Of interest is the potential discordant patterns of EG shedding and associations with the endothelial activation biomarkers. Release of Hyaluronan occurs via incompletely understood mechanisms but includes cleavage of the CD44 molecule by MMP-15, as well as by Hyaluronidase and reactive oxygen species^41^. These distinct mechanisms may explain the unexpected finding of a lack of correlation between the Syndecans and Hyaluronan in this study. Replication of this discordance in further studies is an important first step.

There are other reasons for EG biomarkers to increase in circulation that is not specifically related to EG shedding^27^. The conditions of sepsis may upregulate endothelial Syn-1 and Syn-4 expression, which may contribute to circulating concentrations^42^. Further, serum Hyaluronan concentration may be influenced by interstitial washout of tissue Hyaluronan secondary to fluid loading, which may confound the temporal pattern of EG shedding of Hyaluronan. Others have found that Hyaluronan peaks within the first 3–6 h of treatment^17,43^, and therefore this would not be captured at the time points studied. The lack of increase in Hyaluronan at 24 h suggests that an earlier peak may have been missed. These differences may be due to various mechanisms of shedding, sources of EG biomarkers and temporal patterns and explain why relationships between individual EG biomarkers cannot be established at isolated time points.

There was an association between both Syndecan types and VEGFR-1. This molecule, also known as sFlt-1, has previously been found to be associated with organ failure and an accurate predictor of mortality among ED patients with sepsis^44^. In contrast Hyaluronan was not associated with VEGRF-1 but was associated with ET-1 and Ang-2. This finding supports recent data which suggests Ang-2 plays a role in Hyaluronan shedding^41^. It was also interesting to note the positive correlation between Hyaluronan and inflammatory biomarkers; fragmented Hyaluronan is known to be pro-inflammatory and may have driven this association^45^. When examining associations between biomarkers reported in this study, it is important to note that similar associations were identified at T0 and T24. Glycocalyx shedding and endothelial activation is likely on a continuum prior to, and during, treatment of septic shock, and that, despite attempts to adjust for confounding, this conceals any modification by the volume of resuscitation.

Syn-4 has been studied less than Syn-1 as a biomarker of EG shedding. In one study of patients with pneumonia, Syn-4 levels trended downwards over time, as observed in the present study, and had a negative correlation with illness severity^46^. Another study found no difference in Syn-4 concentrations between critically ill ICU patients and healthy controls^47^. The correlation between Syn-1 and Syn-4 is consistent with increased endothelial expression of these glycosaminoglycans in response to circulating cytokines in systemic inflammation in the setting of critical illness^14^. The discordant patterns between T0 and T24 may reflect downregulation of Syn-4 by Syn-1, as has been demonstrated in vitro^48^.

### Meaning of the study and clinical implications

The clinical importance of this study relates to concerns about a possible detrimental effect of exogenously administered fluids, particularly in the setting of septic shock^20^. The question of the optimal approach to haemodynamic resuscitation in septic shock is important due to the high morbidity and mortality of this condition. A clinical trial in African children with sepsis and hypoperfusion found that fluid resuscitation increased mortality due to shock^6,49^. In a preclinical ovine sepsis model, resuscitation with intravenous fluids resulted in a larger subsequent dose of vasopressor to maintain a target blood pressure^18^. EG shedding is one of several mechanisms by which exogenous fluid resuscitation may be detrimental in septic shock. While emerging evidence favours a fluid-sparing or de-resuscitation fluid strategy among ICU patients with sepsis^4^, there is uncertainty about the optimal approach to initial haemodynamic resuscitation. Our findings do not support the hypothesis that IV fluid resuscitation exacerbates EG shedding in sepsis. Indeed, recent clinical observational studies have suggested that early intravenous fluid resuscitation is associated with reduced odds of mortality among ED patients with septic shock^50,51^. Thus, an overly restrictive fluid strategy in sepsis resuscitation due to fears of causing EG injury may be unwarranted. This is the subject of several active clinical trials^52–54^.

### Unanswered questions and future research

In addition to the impact of IV fluids, further research is required to confirm the apparent finding of different patterns of EG shedding and the implications for clinical practice. The significant heterogeneity of sepsis may make a straightforward answer elusive. Indeed, it is possible that the inability to reproduce the findings of preclinical and healthy volunteer studies in the clinical setting likely reflects the fact that EG shedding is already established in critical illness. Novel therapies directed at restoring the shed glycocalyx is one avenue of current investigation^55^. While the results of clinical trials are awaited, a judicious approach to fluid administration which aims to protect the EG remains prudent^15^.

In conclusion, distinct patterns of association between biomarkers of EG shedding and endothelial cell activation were observed among patients undergoing resuscitation for sepsis. We did not find evidence to support the hypothesis that degradation of the EG is exacerbated by higher resuscitation fluids volumes in patients with septic shock.

## Supplementary Information

Supplementary Information.

## Data Availability

The datasets used in the analyses reported in this paper are available from the corresponding author on reasonable request.
