# Supplementary material for: No association between intravenous fluid volume and endothelial glycocalyx shedding in patients undergoing resuscitation for sepsis in the emergency department
Source: Sci Rep. 2022 May 24;12:8733. doi: 10.1038/s41598-022-12752-x (PMC9130214; doi:10.1038/s41598-022-12752-x)
Supplement: Supplementary file 1 — Supplementary Information. [file 41598_2022_12752_MOESM1_ESM.docx]

# Summary

## Renal sofa score >=3 vs <3

T0: no differences were detected between renal sofa >=3 and <3 for ln_Syndecan1 (p=0.70) ln_syndecan4 (p=0.46) or ln_Hyaluronan (p=0.99).

T24: no differences were detected between renal sofa >=3 and <3 for ln_Syndecan1 (p=0.38) ln_syndecan4 (p=0.12) or ln_Hyaluronan (p=0.07).

## Source of infection

T0: ln_Syndecan1 (p=0.85) ln_Syndecan4 (p=0.31) ln_Hyaluronan (p=0.11)

T24: ln_Syndecan1 (p=0.75) ln_Syndecan4 (p=0.71) ln_Hyaluronan (p=0.06). Pairwise test for UTI vs Respiratory (p=0.018) but the non significant overall test suggest that the pairwise comparisons should be ignored. The overall test is borderline so in an exploratory analysis, would be given some further consideration.

## ICU Admission

T0: ln_Syndecan1 (p=0.07) ln_Syndecan4 (p=0.64) ln_Hyaluronan (p=0.12)

T24: ln_Syndecan1 (p=0.045) ln_Syndecan4 (p=0.40) ln_Hyaluronan (p=0.001).

Given the difference detected in the biomarkers between those admitted to ICU and those not, the interaction of fluids and admission were tested at T24 for ln_Syndecan1 (p=0.095) and ln_hyaluronan (p=0.28) but neither were significant.

## Ventilated

T0: ln_Syndecan1 (p=0.23) ln_Syndecan4 (p=0.54) ln_Hyaluronan (p=0.4)

T24: ln_Syndecan1 (p=0.16) ln_Syndecan4 (p=0.73) ln_Hyaluronan (p=0.047).

Interaction of ventilated and fluids tested at T24 for ln_hyaluronan (p=0.58)

# Sofa renal score (EDRENAL)

## Time 0

. tab1 sofa_renal renal_sofa_ge3 if time==0

-> tabulation of sofa_renal if time==0

sofa_renal | Freq. Percent Cum.

------------+-----------------------------------

0 | 28 32.56 32.56

1 | 23 26.74 59.30

2 | 19 22.09 81.40

3 | 9 10.47 91.86

4 | 7 8.14 100.00

------------+-----------------------------------

Total | 86 100.00

Combined as 3 & 4 vs the rest:

-> tabulation of **renal_sofa_ge3** if time==0

renal_sofa_ |

ge3 | Freq. Percent Cum.

------------+-----------------------------------

0 | 70 81.40 81.40

1 | 16 18.60 100.00

------------+-----------------------------------

Total | 86 100.00

. tabstat Syn1pgml ln_Syndecan1 Syndecan4 ln_Syndecan4 Hyaluronan ln_Hyaluronan ///

> if time==0, by(renal_sofa_ge3) s(n mean sd median min max p25 p75) ///

> col(stats) long f(%8.3g)

renal_sofa_ge3 Variable | N Mean SD p50 Min Max p25 p75

----------------------------+---------------------------------------------------------------

0 Syn1pgml | 69 37617 89644 8002 125 480001 4160 24320

**ln_Syndecan1 | 69 9.3 1.5 8.99 4.83 13.1 8.33 10.1**

Syndecan4 | 66 8182 21110 870 62.5 80001 358 2783

**ln_Syndecan4 | 66 6.97 1.96 6.77 4.14 11.3 5.88 7.93**

Hyaluronan | 70 621 1192 172 .37 7021 64.3 521

**ln_Hyaluro~n | 70 4.9 2.36 5.15 -.994 8.86 4.16 6.26**

----------------------------+---------------------------------------------------------------

1 Syn1pgml | 16 15788 22011 10406 2684 91398 4939 14368

**ln_Syndecan1 | 16 9.17 .935 9.25 7.9 11.4 8.5 9.57**

Syndecan4 | 16 2824 6955 599 62.5 27742 377 1233

**ln_Syndecan4 | 16 6.57 1.51 6.39 4.14 10.2 5.93 7.12**

Hyaluronan | 16 536 1007 147 .37 3861 48.3 365

**ln_Hyaluro~n | 16 4.87 2.12 4.99 -.994 8.26 3.87 5.85**

----------------------------+---------------------------------------------------------------

Total Syn1pgml | 85 33508 81643 8684 125 480001 4412 20883

ln_Syndecan1 | 85 9.28 1.41 9.07 4.83 13.1 8.39 9.95

Syndecan4 | 82 7137 19265 794 62.5 80001 371 1942

ln_Syndecan4 | 82 6.89 1.88 6.68 4.14 11.3 5.91 7.57

Hyaluronan | 86 605 1155 168 .37 7021 63.8 474

ln_Hyaluro~n | 86 4.89 2.31 5.12 -.994 8.86 4.16 6.16

--------------------------------------------------------------------------------------------

. tobit ln_Syndecan1 i.renal_sofa_ge3 if time==0 , ll(ln(125)) ul(ln(480001)) ///

> nolog vce(bootstrap, reps(500) seed(010967))

(running tobit on estimation sample)

Tobit regression Number of obs = 85

Uncensored = 83

Limits: Lower = 4.83 Left-censored = 2

Upper = 13.08 Right-censored = 0

Replications = 500

Wald chi2(1) = 0.15

Prob > chi2 = 0.7014

Log likelihood = -150.9268 Pseudo R2 = 0.0003

-------------------------------------------------------------------------------------

| Observed Bootstrap Normal-based

ln_Syndecan1 | coefficient std. err. z P>|z| [95% conf. interval]

--------------------+----------------------------------------------------------------

1.renal_sofa_ge3 | -.1171235 .3054442 -0.38 0.701 -.7157832 .4815362

_cons | 9.288461 .1899333 48.90 0.000 8.916199 9.660724

--------------------+----------------------------------------------------------------

var(e.ln_Syndecan1)| 2.047087 .4994233 1.269026 3.30219

-------------------------------------------------------------------------------------

. tobit ln_Syndecan4 i.renal_sofa_ge3 if time==0 , ll(ln(62.5)) ul(ln(80000)) ///

> nolog vce(bootstrap, seed(010967) reps(500))

(running tobit on estimation sample)

Tobit regression Number of obs = 82

Uncensored = 64

Limits: Lower = 4.14 Left-censored = 13

Upper = 11.29 Right-censored = 5

Replications = 500

Wald chi2(1) = 0.54

Prob > chi2 = 0.4625

Log likelihood = -166.28402 Pseudo R2 = 0.0013

-------------------------------------------------------------------------------------

| Observed Bootstrap Normal-based

ln_Syndecan4 | coefficient std. err. z P>|z| [95% conf. interval]

--------------------+----------------------------------------------------------------

1.renal_sofa_ge3 | -.4239181 .5770216 -0.73 0.463 -1.55486 .7070235

_cons | 6.845315 .3047824 22.46 0.000 6.247953 7.442678

--------------------+----------------------------------------------------------------

var(e.ln_Syndecan4)| 5.386199 1.398625 3.237816 8.960092

-------------------------------------------------------------------------------------

. tobit ln_Hyaluronan i.renal_sofa_ge3 if time==0 , ll(ln(.37)) ul(ln(7020)) ///

> nolog vce(bootstrap, seed(010967) reps(500))

(running tobit on estimation sample)

Tobit regression Number of obs = 86

Uncensored = 77

Limits: Lower = -0.99 Left-censored = 8

Upper = 8.86 Right-censored = 1

Replications = 500

Wald chi2(1) = 0.00

Prob > chi2 = 0.9863

Log likelihood = -194.34815 Pseudo R2 = 0.0000

--------------------------------------------------------------------------------------

| Observed Bootstrap Normal-based

ln_Hyaluronan | coefficient std. err. z P>|z| [95% conf. interval]

---------------------+----------------------------------------------------------------

1.renal_sofa_ge3 | -.0114678 .6669609 -0.02 0.986 -1.318687 1.295752

_cons | 4.824436 .3467438 13.91 0.000 4.14483 5.504041

---------------------+----------------------------------------------------------------

var(e.ln_Hyaluronan)| 6.464973 1.746252 3.807579 10.97702

--------------------------------------------------------------------------------------

### Time 24

. tab1 sofa_renal renal_sofa_ge3 if time==24

-> tabulation of sofa_renal if time==24

sofa_renal | Freq. Percent Cum.

------------+-----------------------------------

0 | 26 30.23 30.23

1 | 22 25.58 55.81

2 | 9 10.47 66.28

3 | 12 13.95 80.23

4 | 17 19.77 100.00

------------+-----------------------------------

Total | 86 100.00

-> tabulation of renal_sofa_ge3 if time==24

renal_sofa_ |

ge3 | Freq. Percent Cum.

------------+-----------------------------------

0 | 57 66.28 66.28

1 | 29 33.72 100.00

------------+-----------------------------------

Total | 86 100.00

. tabstat Syn1pgml ln_Syndecan1 Syndecan4 ln_Syndecan4 Hyaluronan ln_Hyaluronan ///

> if time==24, by(renal_sofa_ge3) s(n mean sd median min max p25 p75) ///

> col(stats) long f(%8.3g)

renal_sofa_ge3 Variable | N Mean SD p50 Min Max p25 p75

----------------------------+---------------------------------------------------------------

0 Syn1pgml | 57 42406 108066 10273 125 480001 5406 21840

**ln_Syndecan1 | 57 9.36 1.5 9.24 4.83 13.1 8.6 9.99**

Syndecan4 | 54 7895 21081 724 62.5 80001 237 1809

**ln_Syndecan4 | 54 6.78 1.96 6.58 4.14 11.3 5.47 7.5**

Hyaluronan | 57 616 1430 146 .37 7021 40.5 367

**ln_Hyaluro~n | 57 4.41 2.71 4.98 -.994 8.86 3.7 5.9**

----------------------------+---------------------------------------------------------------

1 Syn1pgml | 29 29492 48351 12115 3860 195151 6899 24040

**ln_Syndecan1 | 29 9.59 1.07 9.4 8.26 12.2 8.84 10.1**

Syndecan4 | 29 4487 15171 494 62.5 80001 62.5 1139

**ln_Syndecan4 | 29 6.15 1.93 6.2 4.14 11.3 4.14 7.04**

Hyaluronan | 29 813 1202 321 .37 5291 137 917

**ln_Hyaluro~n | 29 5.52 2.18 5.77 -.994 8.57 4.92 6.82**

----------------------------+---------------------------------------------------------------

Total Syn1pgml | 86 38052 92205 10563 125 480001 5600 23460

ln_Syndecan1 | 86 9.44 1.37 9.26 4.83 13.1 8.63 10.1

Syndecan4 | 83 6704 19197 630 62.5 80001 193 1521

ln_Syndecan4 | 83 6.56 1.96 6.45 4.14 11.3 5.26 7.33

Hyaluronan | 86 683 1354 223 .37 7021 48.4 527

ln_Hyaluro~n | 86 4.78 2.58 5.41 -.994 8.86 3.88 6.27

--------------------------------------------------------------------------------------------

.

. tobit ln_Syndecan1 i.renal_sofa_ge3 if time==24 , ll(ln(125)) ul(ln(480001)) ///

> nolog vce(bootstrap, reps(500) seed(010967))

(running tobit on estimation sample)

Tobit regression Number of obs = 86

Uncensored = 84

Limits: Lower = 4.83 Left-censored = 2

Upper = 13.08 Right-censored = 0

Replications = 500

Wald chi2(1) = 0.77

Prob > chi2 = 0.3813

Log likelihood = -149.97846 Pseudo R2 = 0.0019

-------------------------------------------------------------------------------------

| Observed Bootstrap Normal-based

ln_Syndecan1 | coefficient std. err. z P>|z| [95% conf. interval]

--------------------+----------------------------------------------------------------

1.renal_sofa_ge3 | .2417455 .2761309 0.88 0.381 -.2994612 .7829522

_cons | 9.346075 .2073468 45.07 0.000 8.939683 9.752467

--------------------+----------------------------------------------------------------

var(e.ln_Syndecan1)| 1.91526 .5038319 1.143692 3.207351

-------------------------------------------------------------------------------------

. tobit ln_Syndecan4 i.renal_sofa_ge3 if time==24 , ll(ln(62.5)) ul(ln(80000)) ///

> nolog vce(bootstrap, seed(010967) reps(500))

(running tobit on estimation sample)

Tobit regression Number of obs = 83

Uncensored = 59

Limits: Lower = 4.14 Left-censored = 19

Upper = 11.29 Right-censored = 5

Replications = 500

Wald chi2(1) = 2.38

Prob > chi2 = 0.1231

Log likelihood = -165.5029 Pseudo R2 = 0.0078

-------------------------------------------------------------------------------------

| Observed Bootstrap Normal-based

ln_Syndecan4 | coefficient std. err. z P>|z| [95% conf. interval]

--------------------+----------------------------------------------------------------

1.renal_sofa_ge3 | -1.000013 .6485205 -1.54 0.123 -2.27109 .2710636

_cons | 6.621458 .3509585 18.87 0.000 5.933592 7.309324

--------------------+----------------------------------------------------------------

var(e.ln_Syndecan4)| 6.710186 1.729685 4.048744 11.12113

-------------------------------------------------------------------------------------

. tobit ln_Hyaluronan i.renal_sofa_ge3 if time==24 , ll(ln(.37)) ul(ln(7020)) ///

> nolog vce(bootstrap, seed(010967) reps(500))

(running tobit on estimation sample)

Tobit regression Number of obs = 86

Uncensored = 73

Limits: Lower = -0.99 Left-censored = 11

Upper = 8.86 Right-censored = 2

Replications = 500

Wald chi2(1) = 3.31

Prob > chi2 = 0.0689

Log likelihood = -199.5506 Pseudo R2 = 0.0075

--------------------------------------------------------------------------------------

| Observed Bootstrap Normal-based

ln_Hyaluronan | coefficient std. err. z P>|z| [95% conf. interval]

---------------------+----------------------------------------------------------------

1.renal_sofa_ge3 | 1.175531 .6461874 1.82 0.069 -.090973 2.442035

_cons | 4.269669 .4751044 8.99 0.000 3.338482 5.200857

---------------------+----------------------------------------------------------------

var(e.ln_Hyaluronan)| 8.553983 2.275347 5.07867 14.40744

--------------------------------------------------------------------------------------

# Source of Infection

. tab1 sourcecode if time==0

-> tabulation of sourcecode if time==0

RECODE of |

Sourcecode |

(Source code) | Freq. Percent Cum.

---------------+-----------------------------------

pulmonary/resp | 35 40.70 40.70

UTI | 17 19.77 60.47

other | 34 39.53 100.00

---------------+-----------------------------------

Total | 86 100.00

## T0

. tabstat Syn1pgml ln_Syndecan1 Syndecan4 ln_Syndecan4 Hyaluronan ln_Hyaluronan ///

> if time==0, by(sourcecode) s(n mean sd median min max p25 p75) ///

> col(stats) long f(%8.3g)

sourcecode Variable | N Mean SD p50 Min Max p25 p75

----------------------------+-------------------------------------------------------------

pulmonary/resp Syn1pgml |34 37353 91102 10915 125 480001 4910 22195

**ln_Syndecan1 |34 9.39 1.46 9.3 4.83 13.1 8.5 10**

Syndecan4 |33 4331 14092 701 62.5 80001 383 1362

**ln_Syndecan4 |33 6.61 1.75 6.55 4.14 11.3 5.95 7.22**

Hyaluronan |35 763 1518 151 .37 7021 70.6 716

**ln_Hyaluro~n |35 5 2.35 5.02 -.994 8.86 4.26 6.57**

----------------------------+-------------------------------------------------------------

UTI Syn1pgml |17 27607 54081 6200 2608 217047 3714 12865

**ln_Syndecan1 |17 9.15 1.34 8.73 7.87 12.3 8.22 9.46**

Syndecan4 |16 9317 20279 962 62.5 80001 620 6183

**ln_Syndecan4 |16 7.46 1.96 6.84 4.14 11.3 6.43 8.7**

Hyaluronan |17 197 287 145 .37 1211 42.3 231

**ln_Hyaluro~n |17 3.68 2.8 4.98 -.994 7.1 3.74 5.44**

----------------------------+-------------------------------------------------------------

other Syn1pgml |34 32613 84943 7567 125 480001 5338 16360

**ln_Syndecan1 |34 9.23 1.43 8.93 4.83 13.1 8.58 9.7**

Syndecan4 |33 8885 23118 852 62.5 80001 358 1859

**ln_Syndecan4 |33 6.9 1.96 6.75 4.14 11.3 5.88 7.53**

Hyaluronan |34 647 952 215 .37 3861 81 705

**ln_Hyaluro~n |34 5.38 1.79 5.37 -.994 8.26 4.39 6.56**

----------------------------+-------------------------------------------------------------

Total Syn1pgml |85 33508 81643 8684 125 480001 4412 20883

ln_Syndecan1 |85 9.28 1.41 9.07 4.83 13.1 8.39 9.95

Syndecan4 |82 7137 19265 794 62.5 80001 371 1942

ln_Syndecan4 |82 6.89 1.88 6.68 4.14 11.3 5.91 7.57

Hyaluronan |86 605 1155 168 .37 7021 63.8 474

ln_Hyaluro~n |86 4.89 2.31 5.12 -.994 8.86 4.16 6.16

------------------------------------------------------------------------------------------

. tobit ln_Syndecan1 i.sourcecode if time==0 , ll(ln(125)) ul(ln(480001)) ///

> nolog vce(bootstrap, reps(500) seed(010967))

(running tobit on estimation sample)

Tobit regression Number of obs = 85

Uncensored = 83

Limits: Lower = 4.83 Left-censored = 2

Upper = 13.08 Right-censored = 0

Replications = 500

Wald chi2(2) = 0.32

Prob > chi2 = 0.8513

Log likelihood = -150.79972 Pseudo R2 = 0.0011

-------------------------------------------------------------------------------------

| Observed Bootstrap Normal-based

ln_Syndecan1 | coefficient std. err. z P>|z| [95% conf. interval]

--------------------+----------------------------------------------------------------

sourcecode |

UTI | -.2202124 .4452879 -0.49 0.621 -1.092961 .6525359

other | -.1588822 .3466394 -0.46 0.647 -.8382829 .5205186

|

_cons | 9.374044 .2647513 35.41 0.000 8.855141 9.892947

--------------------+----------------------------------------------------------------

var(e.ln_Syndecan1)| 2.040615 .5011686 1.260982 3.302277

-------------------------------------------------------------------------------------

. tobit ln_Syndecan4 i.sourcecode if time==0 , ll(ln(62.5)) ul(ln(80000)) ///

> nolog vce(bootstrap, seed(010967) reps(500))

(running tobit on estimation sample)

Tobit regression Number of obs = 82

Uncensored = 64

Limits: Lower = 4.14 Left-censored = 13

Upper = 11.29 Right-censored = 5

Replications = 500

Wald chi2(2) = 2.31

Prob > chi2 = 0.3144

Log likelihood = -165.35174 Pseudo R2 = 0.0069

-------------------------------------------------------------------------------------

| Observed Bootstrap Normal-based

ln_Syndecan4 | coefficient std. err. z P>|z| [95% conf. interval]

--------------------+----------------------------------------------------------------

sourcecode |

UTI | 1.072267 .7054292 1.52 0.129 -.3103486 2.454883

other | .4249537 .6433927 0.66 0.509 -.8360728 1.68598

|

_cons | 6.379547 .4114467 15.51 0.000 5.573126 7.185968

--------------------+----------------------------------------------------------------

var(e.ln_Syndecan4)| 5.269704 1.341005 3.200201 8.677513

-------------------------------------------------------------------------------------

. tobit ln_Hyaluronan i.sourcecode if time==0 , ll(ln(.37)) ul(ln(7020)) ///

> nolog vce(bootstrap, seed(010967) reps(500))

(running tobit on estimation sample)

Tobit regression Number of obs = 86

Uncensored = 77

Limits: Lower = -0.99 Left-censored = 8

Upper = 8.86 Right-censored = 1

Replications = 500

Wald chi2(2) = 4.48

Prob > chi2 = 0.1066

Log likelihood = -191.00344 Pseudo R2 = 0.0172

--------------------------------------------------------------------------------------

| Observed Bootstrap Normal-based

ln_Hyaluronan | coefficient std. err. z P>|z| [95% conf. interval]

---------------------+----------------------------------------------------------------

sourcecode |

UTI | -1.509842 .8956924 -1.69 0.092 -3.265366 .2456833

other | .3924409 .5366694 0.73 0.465 -.6594118 1.444294

|

_cons | 4.964264 .4543141 10.93 0.000 4.073825 5.854704

---------------------+----------------------------------------------------------------

var(e.ln_Hyaluronan)| 5.980607 1.508378 3.648064 9.804559

--------------------------------------------------------------------------------------

## T24

. tabstat Syn1pgml ln_Syndecan1 Syndecan4 ln_Syndecan4 Hyaluronan ln_Hyaluronan ///

> if time==24, by(sourcecode) s(n mean sd median min max p25 p75) ///

> col(stats) long f(%8.3g)

sourcecode Variable | N Mean SD p50 Min Max p25 p75

----------------------------+---------------------------------------------------------

pulmonary/resp Syn1pgml | 35 37998 85241 14124 125 480001 6403 27839

**ln_Syndecan1 | 35 9.57 1.4 9.56 4.83 13.1 8.76 10.2**

Syndecan4 | 33 6200 19169 599 62.5 80001 62.5 1420

**ln_Syndecan4 | 33 6.45 1.99 6.4 4.14 11.3 4.14 7.26**

Hyaluronan | 35 775 1407 260 .37 7021 74.3 825

**ln_Hyaluro~n | 35 5.35 2.09 5.56 -.994 8.86 4.31 6.72**

----------------------------+---------------------------------------------------------

UTI Syn1pgml | 17 25106 47836 8584 3780 195151 4920 16270

**ln_Syndecan1 | 17 9.31 1.11 9.06 8.24 12.2 8.5 9.7**

Syndecan4 | 17 7618 19601 847 62.5 80001 281 1858

**ln_Syndecan4 | 17 6.92 2.02 6.74 4.14 11.3 5.64 7.53**

Hyaluronan | 17 171 243 105 .37 1007 26.5 224

**ln_Hyaluro~n | 17 3.54 2.74 4.66 -.994 6.91 3.28 5.41**

----------------------------+---------------------------------------------------------

other Syn1pgml | 34 44579 114772 10067 125 480001 5600 16430

**ln_Syndecan1 | 34 9.36 1.46 9.22 4.83 13.1 8.63 9.71**

Syndecan4 | 33 6737 19598 547 62.5 80001 236 1139

**ln_Syndecan4 | 33 6.47 1.94 6.3 4.14 11.3 5.47 7.04**

Hyaluronan | 34 843 1575 248 .37 7021 44.9 655

**ln_Hyaluro~n | 34 4.82 2.81 5.51 -.994 8.86 3.8 6.48**

----------------------------+---------------------------------------------------------

Total Syn1pgml | 86 38052 92205 10563 125 480001 5600 23460

ln_Syndecan1 | 86 9.44 1.37 9.26 4.83 13.1 8.63 10.1

Syndecan4 | 83 6704 19197 630 62.5 80001 193 1521

ln_Syndecan4 | 83 6.56 1.96 6.45 4.14 11.3 5.26 7.33

Hyaluronan | 86 683 1354 223 .37 7021 48.4 527

ln_Hyaluro~n | 86 4.78 2.58 5.41 -.994 8.86 3.88 6.27

--------------------------------------------------------------------------------------

.

. tobit ln_Syndecan1 i.sourcecode if time==24 , ll(ln(125)) ul(ln(480001)) ///

> nolog vce(bootstrap, reps(500) seed(010967))

(running tobit on estimation sample)

Tobit regression Number of obs = 86

Uncensored = 84

Limits: Lower = 4.83 Left-censored = 2

Upper = 13.08 Right-censored = 0

Replications = 500

Wald chi2(2) = 0.58

Prob > chi2 = 0.7473

Log likelihood = -149.99474 Pseudo R2 = 0.0018

-------------------------------------------------------------------------------------

| Observed Bootstrap Normal-based

ln_Syndecan1 | coefficient std. err. z P>|z| [95% conf. interval]

--------------------+----------------------------------------------------------------

sourcecode |

UTI | -.2494734 .3638144 -0.69 0.493 -.9625365 .4635897

other | -.2119294 .3439568 -0.62 0.538 -.8860724 .4622136

|

_cons | 9.560852 .2423482 39.45 0.000 9.085858 10.03585

--------------------+----------------------------------------------------------------

var(e.ln_Syndecan1)| 1.914322 .5064264 1.139811 3.215118

-------------------------------------------------------------------------------------

.

. tobit ln_Syndecan4 i.sourcecode if time==24 , ll(ln(62.5)) ul(ln(80000)) ///

> nolog vce(bootstrap, seed(010967) reps(500))

(running tobit on estimation sample)

Tobit regression Number of obs = 83

Uncensored = 59

Limits: Lower = 4.14 Left-censored = 19

Upper = 11.29 Right-censored = 5

Replications = 500

Wald chi2(2) = 0.71

Prob > chi2 = 0.7014

Log likelihood = -166.46644 Pseudo R2 = 0.0020

-------------------------------------------------------------------------------------

| Observed Bootstrap Normal-based

ln_Syndecan4 | coefficient std. err. z P>|z| [95% conf. interval]

--------------------+----------------------------------------------------------------

sourcecode |

UTI | .6397487 .7901765 0.81 0.418 -.9089688 2.188466

other | .1238463 .6747618 0.18 0.854 -1.198663 1.446355

|

_cons | 6.096396 .499244 12.21 0.000 5.117896 7.074896

--------------------+----------------------------------------------------------------

var(e.ln_Syndecan4)| 6.832207 1.786659 4.092298 11.40656

-------------------------------------------------------------------------------------

.

. tobit ln_Hyaluronan i.sourcecode if time==24 , ll(ln(.37)) ul(ln(7020)) ///

> nolog vce(bootstrap, seed(010967) reps(500))

(running tobit on estimation sample)

Tobit regression Number of obs = 86

Uncensored = 73

Limits: Lower = -0.99 Left-censored = 11

Upper = 8.86 Right-censored = 2

Replications = 500

Wald chi2(2) = 5.76

Prob > chi2 = 0.0562

Log likelihood = -198.19461 Pseudo R2 = 0.0142

--------------------------------------------------------------------------------------

| Observed Bootstrap Normal-based

ln_Hyaluronan | coefficient std. err. z P>|z| [95% conf. interval]

---------------------+----------------------------------------------------------------

sourcecode |

UTI | -2.093912 .8869018 -2.36 0.018 -3.832207 -.3556161

other | -.6314313 .6485288 -0.97 0.330 -1.902525 .6396618

|

_cons | 5.331015 .4343227 12.27 0.000 4.479758 6.182272

---------------------+----------------------------------------------------------------

var(e.ln_Hyaluronan)| 8.315685 2.173923 4.98165 13.88107

--------------------------------------------------------------------------------------

# ICU admission

. tab1 ICU_Adm if time==0

-> tabulation of ICU_Adm if time==0

ICU_Adm | Freq. Percent Cum.

------------+-----------------------------------

0 | 21 24.42 24.42

1 | 65 75.58 100.00

------------+-----------------------------------

Total | 86 100.00

## T0

. tabstat Syn1pgml ln_Syndecan1 Syndecan4 ln_Syndecan4 Hyaluronan ln_Hyaluronan ///

> if time==0, by(ICU_Adm) s(n mean sd median min max p25 p75) ///

> col(stats) long f(%8.3g)

ICU_Adm Variable | N Mean SD p50 Min Max p25 p75

----------------------+--------------------------------------------------------

0 Syn1pgml | 21 12684 12928 7428 125 47920 3720 19928

**ln_Syndecan1 | 21 8.73 1.58 8.91 4.83 10.8 8.22 9.9**

Syndecan4 | 19 2919 4683 954 62.5 19340 377 4614

**ln_Syndecan4 | 19 7 1.48 6.86 4.14 9.87 5.93 8.44**

Hyaluronan | 21 403 818 64.3 .37 3360 35.3 194

**ln_Hyaluro~n | 21 4.11 2.54 4.16 -.994 8.12 3.56 5.27**

----------------------+--------------------------------------------------------

1 Syn1pgml | 64 40340 92965 9079 2684 480001 4939 27081

**ln_Syndecan1 | 64 9.45 1.31 9.11 7.9 13.1 8.5 10.2**

Syndecan4 | 63 8409 21712 774 62.5 80001 341 1942

**ln_Syndecan4 | 63 6.86 1.99 6.65 4.14 11.3 5.83 7.57**

Hyaluronan | 65 671 1242 214 .37 7021 93.3 521

**ln_Hyaluro~n | 65 5.14 2.19 5.37 -.994 8.86 4.54 6.26**

----------------------+--------------------------------------------------------

Total Syn1pgml | 85 33508 81643 8684 125 480001 4412 20883

ln_Syndecan1 | 85 9.28 1.41 9.07 4.83 13.1 8.39 9.95

Syndecan4 | 82 7137 19265 794 62.5 80001 371 1942

ln_Syndecan4 | 82 6.89 1.88 6.68 4.14 11.3 5.91 7.57

Hyaluronan | 86 605 1155 168 .37 7021 63.8 474

ln_Hyaluro~n | 86 4.89 2.31 5.12 -.994 8.86 4.16 6.16

-------------------------------------------------------------------------------

. tobit ln_Syndecan1 i.ICU_Adm if time==0 , ll(ln(125)) ul(ln(480001)) ///

> nolog vce(bootstrap, reps(500) seed(010967))

(running tobit on estimation sample)

Tobit regression Number of obs = 85

Uncensored = 83

Limits: Lower = 4.83 Left-censored = 2

Upper = 13.08 Right-censored = 0

Replications = 500

Wald chi2(1) = 3.28

Prob > chi2 = 0.0701

Log likelihood = -148.69783 Pseudo R2 = 0.0151

-------------------------------------------------------------------------------------

| Observed Bootstrap Normal-based

ln_Syndecan1 | coefficient std. err. z P>|z| [95% conf. interval]

--------------------+----------------------------------------------------------------

1.ICU_Adm | .7590726 .4190386 1.81 0.070 -.062228 1.580373

_cons | 8.694277 .3894993 22.32 0.000 7.930872 9.457681

--------------------+----------------------------------------------------------------

var(e.ln_Syndecan1)| 1.948184 .4023636 1.299658 2.920323

-------------------------------------------------------------------------------------

. tobit ln_Syndecan4 i.ICU_Adm if time==0 , ll(ln(62.5)) ul(ln(80000)) ///

> nolog vce(bootstrap, seed(010967) reps(500))

(running tobit on estimation sample)

Tobit regression Number of obs = 82

Uncensored = 64

Limits: Lower = 4.14 Left-censored = 13

Upper = 11.29 Right-censored = 5

Replications = 500

Wald chi2(1) = 0.22

Prob > chi2 = 0.6405

Log likelihood = -166.42153 Pseudo R2 = 0.0004

-------------------------------------------------------------------------------------

| Observed Bootstrap Normal-based

ln_Syndecan4 | coefficient std. err. z P>|z| [95% conf. interval]

--------------------+----------------------------------------------------------------

1.ICU_Adm | -.2313299 .495381 -0.47 0.641 -1.202259 .739599

_cons | 6.937257 .3799587 18.26 0.000 6.192552 7.681962

--------------------+----------------------------------------------------------------

var(e.ln_Syndecan4)| 5.416499 1.42648 3.232567 9.0759

-------------------------------------------------------------------------------------

. tobit ln_Hyaluronan i.ICU_Adm if time==0 , ll(ln(.37)) ul(ln(7020)) ///

> nolog vce(bootstrap, seed(010967) reps(500))

(running tobit on estimation sample)

Tobit regression Number of obs = 86

Uncensored = 77

Limits: Lower = -0.99 Left-censored = 8

Upper = 8.86 Right-censored = 1

Replications = 500

Wald chi2(1) = 2.36

Prob > chi2 = 0.1244

Log likelihood = -192.77988 Pseudo R2 = 0.0081

--------------------------------------------------------------------------------------

| Observed Bootstrap Normal-based

ln_Hyaluronan | coefficient std. err. z P>|z| [95% conf. interval]

---------------------+----------------------------------------------------------------

1.ICU_Adm | 1.126081 .7327612 1.54 0.124 -.3101048 2.562266

_cons | 3.972245 .6643285 5.98 0.000 2.670185 5.274305

---------------------+----------------------------------------------------------------

var(e.ln_Hyaluronan)| 6.21796 1.688079 3.652246 10.5861

--------------------------------------------------------------------------------------

## T24

. tabstat Syn1pgml ln_Syndecan1 Syndecan4 ln_Syndecan4 Hyaluronan ln_Hyaluronan ///

> if time==24, by(ICU_Adm) s(n mean sd median min max p25 p75) ///

> col(stats) long f(%8.3g)

ICU_Adm Variable | N Mean SD p50 Min Max p25 p75

----------------------+-------------------------------------------------------------

0 Syn1pgml | 21 13272 12553 8988 125 50200 4688 19376

**ln_Syndecan1 | 21 8.86 1.55 9.1 4.83 10.8 8.45 9.87**

Syndecan4 | 19 5542 18127 736 62.5 80001 335 1858

**ln_Syndecan4 | 19 6.77 1.67 6.6 4.14 11.3 5.81 7.53**

Hyaluronan | 21 151 269 49.6 .37 1037 .37 136

**ln_Hyaluro~n | 21 2.69 3.07 3.9 -.994 6.94 -.994 4.91**

----------------------+-------------------------------------------------------------

1 Syn1pgml | 65 46057 104765 10830 2680 480001 6403 24040

**ln_Syndecan1 | 65 9.62 1.26 9.29 7.89 13.1 8.76 10.1**

Syndecan4 | 64 7049 19627 615 62.5 80001 62.5 1436

**ln_Syndecan4 | 64 6.49 2.05 6.42 4.14 11.3 4.14 7.27**

Hyaluronan | 65 854 1513 267 .37 7021 109 825

**ln_Hyaluro~n | 65 5.46 2.01 5.59 -.994 8.86 4.69 6.72**

----------------------+-------------------------------------------------------------

Total Syn1pgml | 86 38052 92205 10563 125 480001 5600 23460

ln_Syndecan1 | 86 9.44 1.37 9.26 4.83 13.1 8.63 10.1

Syndecan4 | 83 6704 19197 630 62.5 80001 193 1521

ln_Syndecan4 | 83 6.56 1.96 6.45 4.14 11.3 5.26 7.33

Hyaluronan | 86 683 1354 223 .37 7021 48.4 527

ln_Hyaluro~n | 86 4.78 2.58 5.41 -.994 8.86 3.88 6.27

------------------------------------------------------------------------------------

. tobit ln_Syndecan1 i.ICU_Adm if time==24 , ll(ln(125)) ul(ln(480001)) ///

> nolog vce(bootstrap, reps(500) seed(010967))

(running tobit on estimation sample)

Tobit regression Number of obs = 86

Uncensored = 84

Limits: Lower = 4.83 Left-censored = 2

Upper = 13.08 Right-censored = 0

Replications = 500

Wald chi2(1) = 4.00

Prob > chi2 = 0.0454

Log likelihood = -147.5523 Pseudo R2 = 0.0181

-------------------------------------------------------------------------------------

| Observed Bootstrap Normal-based

ln_Syndecan1 | coefficient std. err. z P>|z| [95% conf. interval]

--------------------+----------------------------------------------------------------

1.ICU_Adm | .8013726 .4005497 2.00 0.045 .0163096 1.586436

_cons | 8.821504 .366216 24.09 0.000 8.103734 9.539274

--------------------+----------------------------------------------------------------

var(e.ln_Syndecan1)| 1.814014 .4168025 1.156276 2.8459

-------------------------------------------------------------------------------------

. tobit ln_Syndecan1 c.tot_cfluids_MC##i.ICU_Adm if time==24 , ///

> ll(ln(125)) ul(ln(480001)) ///

> nolog vce(bootstrap, reps(500) seed(010967))

(running tobit on estimation sample)

Tobit regression Number of obs = 86

Uncensored = 84

Limits: Lower = 4.83 Left-censored = 2

Upper = 13.08 Right-censored = 0

Replications = 500

Wald chi2(3) = 5.12

Prob > chi2 = 0.1631

Log likelihood = -146.03329 Pseudo R2 = 0.0282

------------------------------------------------------------------------------------------

| Observed Bootstrap Normal-based

ln_Syndecan1 | coefficient std. err. z P>|z| [95% conf. interval]

-------------------------+----------------------------------------------------------------

tot_cfluids_MC | -.3002596 .1968902 -1.53 0.127 -.6861573 .085638

1.ICU_Adm | .9338972 .4452723 2.10 0.036 .0611794 1.806615

|

ICU_Adm#c.tot_cfluids_MC |

1 | .3564403 .2133433 1.67 0.095 -.0617049 .7745855

|

_cons | 8.681291 .4111846 21.11 0.000 7.875384 9.487198

-------------------------+----------------------------------------------------------------

var(e.ln_Syndecan1)| 1.750882 .3959785 1.123957 2.727496

------------------------------------------------------------------------------------------

. tobit ln_Syndecan4 i.ICU_Adm if time==24 , ll(ln(62.5)) ul(ln(80000)) ///

> nolog vce(bootstrap, seed(010967) reps(500))

(running tobit on estimation sample)

Tobit regression Number of obs = 83

Uncensored = 59

Limits: Lower = 4.14 Left-censored = 19

Upper = 11.29 Right-censored = 5

Replications = 500

Wald chi2(1) = 0.72

Prob > chi2 = 0.3972

Log likelihood = -166.51736 Pseudo R2 = 0.0017

-------------------------------------------------------------------------------------

| Observed Bootstrap Normal-based

ln_Syndecan4 | coefficient std. err. z P>|z| [95% conf. interval]

--------------------+----------------------------------------------------------------

1.ICU_Adm | -.5219639 .6165609 -0.85 0.397 -1.730401 .6864733

_cons | 6.676527 .5004073 13.34 0.000 5.695747 7.657307

--------------------+----------------------------------------------------------------

var(e.ln_Syndecan4)| 6.867824 1.793239 4.116842 11.45709

-------------------------------------------------------------------------------------

.

. tobit ln_Hyaluronan i.ICU_Adm if time==24 , ll(ln(.37)) ul(ln(7020)) ///

> nolog vce(bootstrap, seed(010967) reps(500))

(running tobit on estimation sample)

Tobit regression Number of obs = 86

Uncensored = 73

Limits: Lower = -0.99 Left-censored = 11

Upper = 8.86 Right-censored = 2

Replications = 500

Wald chi2(1) = 11.41

Prob > chi2 = 0.0007

Log likelihood = -190.61307 Pseudo R2 = 0.0519

--------------------------------------------------------------------------------------

| Observed Bootstrap Normal-based

ln_Hyaluronan | coefficient std. err. z P>|z| [95% conf. interval]

---------------------+----------------------------------------------------------------

1.ICU_Adm | 3.255471 .9635546 3.38 0.001 1.366938 5.144003

_cons | 2.201529 .9348328 2.35 0.019 .3692902 4.033767

---------------------+----------------------------------------------------------------

var(e.ln_Hyaluronan)| 6.961892 1.642707 4.384099 11.0554

--------------------------------------------------------------------------------------

tobit ln_Hyaluronan c.tot_cfluids_MC##i.ICU_Adm if time==24 , ///

> ll(ln(.37)) ul(ln(7020)) ///

> nolog vce(bootstrap, seed(010967) reps(500))

(running tobit on estimation sample)

Tobit regression Number of obs = 86

Uncensored = 73

Limits: Lower = -0.99 Left-censored = 11

Upper = 8.86 Right-censored = 2

Replications = 500

Wald chi2(3) = 11.48

Prob > chi2 = 0.0094

Log likelihood = -188.67876 Pseudo R2 = 0.0615

------------------------------------------------------------------------------------------

| Observed Bootstrap Normal-based

ln_Hyaluronan | coefficient std. err. z P>|z| [95% conf. interval]

-------------------------+----------------------------------------------------------------

tot_cfluids_MC | -.7788189 .7557463 -1.03 0.303 -2.260054 .7024166

1.ICU_Adm | 3.631306 1.16096 3.13 0.002 1.355866 5.906747

|

ICU_Adm#c.tot_cfluids_MC |

1 | .8213307 .7613795 1.08 0.281 -.6709456 2.313607

|

_cons | 1.819202 1.142915 1.59 0.111 -.4208707 4.059275

-------------------------+----------------------------------------------------------------

var(e.ln_Hyaluronan)| 6.669036 1.561997 4.214032 10.55427

------------------------------------------------------------------------------------------

# Ventilated

. tab1 Ventilated_yn if time==0

-> tabulation of Ventilated_yn if time==0

Ventilated_ |

yn | Freq. Percent Cum.

------------+-----------------------------------

0 | 60 69.77 69.77

1 | 26 30.23 100.00

------------+-----------------------------------

Total | 86 100.00

## T0

.

. tabstat Syn1pgml ln_Syndecan1 Syndecan4 ln_Syndecan4 Hyaluronan ln_Hyaluronan ///

> if time==0, by(Ventilated_yn) s(n mean sd median min max p25 p75) ///

> col(stats) long f(%8.3g)

Ventilated_yn Variable | N Mean SD p50 Min Max p25 p75

---------------------------+---------------------------------------------------------------

0 Syn1pgml | 59 24902 63795 7428 125 480001 3972 20883

**ln_Syndecan1 | 59 9.16 1.37 8.91 4.83 13.1 8.29 9.95**

Syndecan4 | 56 5367 15158 833 62.5 80001 348 3289

**ln_Syndecan4 | 56 6.82 1.85 6.72 4.14 11.3 5.85 8.09**

Hyaluronan | 60 695 1336 174 .37 7021 59.7 450

**ln_Hyaluro~n | 60 5.03 2.16 5.16 -.994 8.86 4.09 6.11**

---------------------------+---------------------------------------------------------------

1 Syn1pgml | 26 53036 111279 10470 2684 480001 4412 22195

**ln_Syndecan1 | 26 9.55 1.47 9.25 7.9 13.1 8.39 10**

Syndecan4 | 26 10949 25978 703 62.5 80001 537 1380

**ln_Syndecan4 | 26 7.04 1.97 6.55 4.14 11.3 6.29 7.23**

Hyaluronan | 26 399 510 154 .37 1820 70.6 521

**ln_Hyaluro~n | 26 4.57 2.64 5.04 -.994 7.51 4.26 6.26**

---------------------------+---------------------------------------------------------------

Total Syn1pgml | 85 33508 81643 8684 125 480001 4412 20883

ln_Syndecan1 | 85 9.28 1.41 9.07 4.83 13.1 8.39 9.95

Syndecan4 | 82 7137 19265 794 62.5 80001 371 1942

ln_Syndecan4 | 82 6.89 1.88 6.68 4.14 11.3 5.91 7.57

Hyaluronan | 86 605 1155 168 .37 7021 63.8 474

ln_Hyaluro~n | 86 4.89 2.31 5.12 -.994 8.86 4.16 6.16

-------------------------------------------------------------------------------------------

. tobit ln_Syndecan1 i.Ventilated_yn if time==0 , ll(ln(125)) ul(ln(480001)) ///

> nolog vce(bootstrap, reps(500) seed(010967))

(running tobit on estimation sample)

Tobit regression Number of obs = 85

Uncensored = 83

Limits: Lower = 4.83 Left-censored = 2

Upper = 13.08 Right-censored = 0

Replications = 500

Wald chi2(1) = 1.44

Prob > chi2 = 0.2308

Log likelihood = -150.24285 Pseudo R2 = 0.0048

-------------------------------------------------------------------------------------

| Observed Bootstrap Normal-based

ln_Syndecan1 | coefficient std. err. z P>|z| [95% conf. interval]

--------------------+----------------------------------------------------------------

1.Ventilated_yn | .4048278 .3378284 1.20 0.231 -.2573037 1.066959

_cons | 9.142478 .1900031 48.12 0.000 8.770079 9.514877

--------------------+----------------------------------------------------------------

var(e.ln_Syndecan1)| 2.015484 .4642918 1.2832 3.165659

-------------------------------------------------------------------------------------

. tobit ln_Syndecan4 i.Ventilated_yn if time==0 , ll(ln(62.5)) ul(ln(80000)) ///

> nolog vce(bootstrap, seed(010967) reps(500))

(running tobit on estimation sample)

Tobit regression Number of obs = 82

Uncensored = 64

Limits: Lower = 4.14 Left-censored = 13

Upper = 11.29 Right-censored = 5

Replications = 500

Wald chi2(1) = 0.38

Prob > chi2 = 0.5373

Log likelihood = -166.27203 Pseudo R2 = 0.0013

-------------------------------------------------------------------------------------

| Observed Bootstrap Normal-based

ln_Syndecan4 | coefficient std. err. z P>|z| [95% conf. interval]

--------------------+----------------------------------------------------------------

1.Ventilated_yn | .3730711 .6047105 0.62 0.537 -.8121397 1.558282

_cons | 6.642658 .3104707 21.40 0.000 6.034146 7.251169

--------------------+----------------------------------------------------------------

var(e.ln_Syndecan4)| 5.406457 1.384767 3.272599 8.931671

-------------------------------------------------------------------------------------

. tobit ln_Hyaluronan i.Ventilated_yn if time==0 , ll(ln(.37)) ul(ln(7020)) ///

> nolog vce(bootstrap, seed(010967) reps(500))

(running tobit on estimation sample)

Tobit regression Number of obs = 86

Uncensored = 77

Limits: Lower = -0.99 Left-censored = 8

Upper = 8.86 Right-censored = 1

Replications = 500

Wald chi2(1) = 0.70

Prob > chi2 = 0.4030

Log likelihood = -193.90597 Pseudo R2 = 0.0023

--------------------------------------------------------------------------------------

| Observed Bootstrap Normal-based

ln_Hyaluronan | coefficient std. err. z P>|z| [95% conf. interval]

---------------------+----------------------------------------------------------------

1.Ventilated_yn | -.5638026 .6741632 -0.84 0.403 -1.885138 .757533

_cons | 4.992434 .3167573 15.76 0.000 4.371601 5.613267

---------------------+----------------------------------------------------------------

var(e.ln_Hyaluronan)| 6.407837 1.688149 3.823506 10.73893

--------------------------------------------------------------------------------------

# T24

. tabstat Syn1pgml ln_Syndecan1 Syndecan4 ln_Syndecan4 Hyaluronan ln_Hyaluronan ///

> if time==24, by(Ventilated_yn) s(n mean sd median min max p25 p75) ///

> col(stats) long f(%8.3g)

Ventilated_yn Variable | N Mean SD p50 Min Max p25 p75

---------------------------+-----------------------------------------------------------

0 Syn1pgml | 60 31899 86028 9616 125 480001 5498 21708

**ln_Syndecan1 | 60 9.3 1.36 9.17 4.83 13.1 8.61 9.99**

Syndecan4 | 57 5908 18061 548 62.5 80001 222 1521

**ln_Syndecan4 | 57 6.49 1.9 6.31 4.14 11.3 5.4 7.33**

Hyaluronan | 60 683 1494 198 .37 7021 39.9 403

**ln_Hyaluro~n | 60 4.47 2.8 5.29 -.994 8.86 3.69 6**

---------------------------+-----------------------------------------------------------

1 Syn1pgml | 26 52250 105544 12553 2680 480001 6320 25603

**ln_Syndecan1 | 26 9.74 1.35 9.44 7.89 13.1 8.75 10.2**

Syndecan4 | 26 8449 21758 636 62.5 80001 193 1450

**ln_Syndecan4 | 26 6.69 2.12 6.45 4.14 11.3 5.26 7.28**

Hyaluronan | 26 681 982 321 .37 3938 109 851

**ln_Hyaluro~n | 26 5.51 1.85 5.77 -.994 8.28 4.69 6.75**

---------------------------+-----------------------------------------------------------

Total Syn1pgml | 86 38052 92205 10563 125 480001 5600 23460

ln_Syndecan1 | 86 9.44 1.37 9.26 4.83 13.1 8.63 10.1

Syndecan4 | 83 6704 19197 630 62.5 80001 193 1521

ln_Syndecan4 | 83 6.56 1.96 6.45 4.14 11.3 5.26 7.33

Hyaluronan | 86 683 1354 223 .37 7021 48.4 527

ln_Hyaluro~n | 86 4.78 2.58 5.41 -.994 8.86 3.88 6.27

---------------------------------------------------------------------------------------

. tobit ln_Syndecan1 i.Ventilated_yn if time==24 , ll(ln(125)) ul(ln(480001)) ///

> nolog vce(bootstrap, reps(500) seed(010967))

(running tobit on estimation sample)

Tobit regression Number of obs = 86

Uncensored = 84

Limits: Lower = 4.83 Left-censored = 2

Upper = 13.08 Right-censored = 0

Replications = 500

Wald chi2(1) = 1.97

Prob > chi2 = 0.1605

Log likelihood = -149.30273 Pseudo R2 = 0.0064

-------------------------------------------------------------------------------------

| Observed Bootstrap Normal-based

ln_Syndecan1 | coefficient std. err. z P>|z| [95% conf. interval]

--------------------+----------------------------------------------------------------

1.Ventilated_yn | .4510514 .321438 1.40 0.161 -.1789554 1.081058

_cons | 9.29127 .1831074 50.74 0.000 8.932386 9.650153

--------------------+----------------------------------------------------------------

var(e.ln_Syndecan1)| 1.884984 .4810894 1.143043 3.108516

-------------------------------------------------------------------------------------

.

. tobit ln_Syndecan4 i.Ventilated_yn if time==24 , ll(ln(62.5)) ul(ln(80000)) ///

> nolog vce(bootstrap, seed(010967) reps(500))

(running tobit on estimation sample)

Tobit regression Number of obs = 83

Uncensored = 59

Limits: Lower = 4.14 Left-censored = 19

Upper = 11.29 Right-censored = 5

Replications = 500

Wald chi2(1) = 0.12

Prob > chi2 = 0.7269

Log likelihood = -166.73024 Pseudo R2 = 0.0004

-------------------------------------------------------------------------------------

| Observed Bootstrap Normal-based

ln_Syndecan4 | coefficient std. err. z P>|z| [95% conf. interval]

--------------------+----------------------------------------------------------------

1.Ventilated_yn | .2335088 .6684814 0.35 0.727 -1.076691 1.543708

_cons | 6.204414 .3517494 17.64 0.000 5.514998 6.89383

--------------------+----------------------------------------------------------------

var(e.ln_Syndecan4)| 6.880986 1.800956 4.119706 11.49305

-------------------------------------------------------------------------------------

.

. tobit ln_Hyaluronan i.Ventilated_yn if time==24 , ll(ln(.37)) ul(ln(7020)) ///

> nolog vce(bootstrap, seed(010967) reps(500))

(running tobit on estimation sample)

Tobit regression Number of obs = 86

Uncensored = 73

Limits: Lower = -0.99 Left-censored = 11

Upper = 8.86 Right-censored = 2

Replications = 500

Wald chi2(1) = 3.96

Prob > chi2 = 0.0465

Log likelihood = -199.66828 Pseudo R2 = 0.0069

--------------------------------------------------------------------------------------

| Observed Bootstrap Normal-based

ln_Hyaluronan | coefficient std. err. z P>|z| [95% conf. interval]

---------------------+----------------------------------------------------------------

1.Ventilated_yn | 1.161396 .5835002 1.99 0.047 .0177568 2.305036

_cons | 4.313648 .4690537 9.20 0.000 3.394319 5.232976

---------------------+----------------------------------------------------------------

var(e.ln_Hyaluronan)| 8.60585 2.240233 5.166698 14.33423

--------------------------------------------------------------------------------------

. tobit ln_Hyaluronan c.tot_cfluids_MC##i.Ventilated_yn if time==24 , ///

> ll(ln(.37)) ul(ln(7020)) ///

> nolog vce(bootstrap, seed(010967) reps(500))

(running tobit on estimation sample)

Tobit regression Number of obs = 86

Uncensored = 73

Limits: Lower = -0.99 Left-censored = 11

Upper = 8.86 Right-censored = 2

Replications = 500

Wald chi2(3) = 6.35

Prob > chi2 = 0.0958

Log likelihood = -199.50603 Pseudo R2 = 0.0077

------------------------------------------------------------------------------------------------

| Observed Bootstrap Normal-based

ln_Hyaluronan | coefficient std. err. z P>|z| [95% conf. interval]

-------------------------------+----------------------------------------------------------------

tot_cfluids_MC | -.0179064 .1162148 -0.15 0.878 -.2456833 .2098706

1.Ventilated_yn | 1.120732 .628625 1.78 0.075 -.1113504 2.352814

|

Ventilated_yn#c.tot_cfluids_MC |

1 | .1609903 .2868619 0.56 0.575 -.4012487 .7232293

|

_cons | 4.311893 .4721706 9.13 0.000 3.386456 5.237331

-------------------------------+----------------------------------------------------------------

var(e.ln_Hyaluronan)| 8.574069 2.188881 5.19856 14.14135

------------------------------------------------------------------------------------------------
